# The ligamentum teres and its role in hip arthroscopy for femoroacetabular impingement: a systematic review

**DOI:** 10.1186/s10195-024-00810-1

**Published:** 2024-12-20

**Authors:** Filippo Migliorini, Federico Cocconi, Tommaso Bardazzi, Virginia Masoni, Virginia Gardino, Gennaro Pipino, Nicola Maffulli

**Affiliations:** 1 Department of Orthopaedic and Trauma Surgery, Academic Hospital of Bolzano (SABES-ASDAA), 39100 Bolzano, Italy; 2 Department of Life Sciences, Health, and Health Professions, Link Campus University, 00165 Rome, Italy; 3 Department of Orthopaedics and Traumatology, University of Turin, Via Zuretti, 29, 10126 Turin, Italy; 4 Department of Orthopaedics and Traumatology, University of Bologna, Via G. Cesare Pupilli, 1, 40136 Bologna, Italy; 5 Department of Orthopaedics, Villa Erbosa Hospital, San Raffaele University, Milan, Italy; 6 Department of Trauma and Orthopaedic Surgery, Faculty of Medicine and Psychology, University La Sapienza, 00185 Rome, Italy; 7 Faculty of Medicine, School of Pharmacy and Bioengineering, Keele University, Stoke on Trent, ST4 7QB UK; 8 Centre for Sports and Exercise Medicine, Barts and the London School of Medicine and Dentistry, Mile End Hospital, Queen Mary University of London, London, E1 4DG UK

**Keywords:** Ligamentum teres, LT, FAI, Hip arthroscopy, Tears, PROMs

## Abstract

**Background:**

The ligamentum teres (LT) has received attention in patients undergoing hip arthroscopy (HA) for femoroacetabular impingement (FAI). Indeed, a better understanding of the function of the LT and its implications for clinical outcomes in the presence of a torn LT is required. This systematic review analyses the patient-reported outcome measures (PROMs) and the complication rate when an intact or torn LT is encountered during HA for FAI.

**Methods:**

Studies that compared patients with an intact to those with a torn LT managed with debridement during hip arthroscopy for FAI were identified from the Web of Science, PubMed, and Embase. The minimum follow-up for inclusion was 24 months. The Risk of Bias in Non-randomised Studies of Interventions (ROBINS-I) tool was used to assess the risk of bias. Patient characteristics and PROMs were assessed at the baseline and last follow-up.

**Results:**

The systematic review identified two studies comprising 611 patients. No statistically significant difference was found in pain, Harris Hip Score, and the activities of daily living and sports subscales of the Hip Outcome Score between patients with an intact LT and those with a torn LT treated with debridement, both of whom underwent HA for FAI.

**Conclusions:**

In patients undergoing arthroscopic management for FAI, an intact or torn ligamentum teres managed with debridement does not influence postoperative PROMs. Given the importance of the LT as a structure of the hip joint and the increasing interest in HA for FAI, further standardised studies will be a valuable source for surgeons encountering this pathology.

**Supplementary Information:**

The online version contains supplementary material available at 10.1186/s10195-024-00810-1.

## Introduction

The ligamentum capitis femoris, commonly called the ligamentum teres (LT), is an anatomical intra-articular extra**-**synovial structure running from the acetabular cotyloid fossa to the fovea on the femoral head [1–4]. Historically, the LT was considered a vestigial structure of the hip; however, it is gaining importance as a secondary stabiliser of the hip joint and plays a vital role in proprioception and nociception [2, 4–7]. The LT is tightly stretched either in maximum flexion and external rotation or when the hip is internally rotated in extension, preventing subluxation of the femoral head at the extreme range of motion [4, 5, 8–12]. The most common LT pathologies are synovitis and partial or complete tears, with a prevalence of up to 90% at hip arthroscopy (HA) [2, 9, 13–16]. Incomplete to complete LT lesions result in the loss of the capacity of the ligament fibres to adsorb and resist mechanical forces, leading to hip joint cartilage damage and hip pain [1, 4, 17]. The major risk factors for developing LT tears are female sex, advanced age, generalised ligamentous laxity, and high-energy activities or direct trauma to the hip [1, 2, 4, 7, 16–19]. Moreover, LT tears can be associated with other hip pathologies such as femoroacetabular impingement (FAI), osteoarthritis, synovial enchondromatosis, osteonecrosis of the femoral head, and hip dysplasia [9, 11, 15, 18–25]. Diagnosis of LT injury is difficult because imaging and physical examination are nonspecific, and differential diagnosis with other hip pathologies is often difficult [26, 27]; magnetic resonance imaging demonstrated a sensitivity and specificity of 50% and 34%, respectively, in identifying any pathological process of the LT [2, 20, 28, 29]. Clinically, a complete hip gait, range of motion, and stability evaluation are required [2]. There is no specific test to assess LT tears [2]. However, the anterior and posterior “shuck” tests to determine the anterior and posterior microinstability and O’Donnell’s test are beneficial [2, 14, 30, 31]. Clinical suspicion is essential until HA confirms the diagnosis [2]. The first strategy to manage LT lesions is conservative and mainly based on low-demand and non-impacting physical activities, core muscle and dynamic hip stabiliser strengthening exercises, painkillers with anti-inflammatory medications, and intra-articular injections [1, 2, 4, 15, 32].

On the contrary, surgical management of LT tears mainly consists of debridement and synovectomy, with some patients requiring reconstruction for persistent symptoms [1, 2, 4, 5, 15, 22, 26, 30, 32–36]. Controversy exists concerning FAI and LT tears since it is uncertain whether FAI is one of the leading causes of degenerative LT tears from the presence of bony prominences or whether LT damage, along with hip microinstability, is a predisposing factor for the worsening of FAI [19, 24, 25, 37, 38]. In addition to the primary surgical procedure for the LT, invasive treatments are indicated when tears are associated with other pathologies [2]. The joint capsule is usually reconstructed or plicated in the case of instability; femoroplasty or acetabuloplasty are advised to remove osseous impingement and osteophytes, and muscular deficiencies such as gluteus medius tears should be managed [2, 5, 15, 30, 32]. In the current literature, systematic reviews reporting results of different treatments for LT tears, such as its debridement or reconstruction, are reported; however, few studies have evaluated whether LT pathology influences the outcomes after hip arthroscopy [2, 15]. This systematic review aims to comprehensively analyse recent evidence concerning the LT and its implication for clinical outcomes when tears or an intact LT are encountered during HA for FAI. Specifically, the main outcomes of interest considered were patient-reported outcome measures (PROMs) and the complication rate at the latest follow-up in both the intact and torn LT groups.

## Methods

### Eligibility criteria

All clinical studies concerning the arthroscopic management of FAI were considered. Only studies that compared two populations of patients—those with a torn and those with an intact ligamentum teres—undergoing comparable surgical procedures were included. Only studies published in peer-reviewed journals were eligible. According to the authors’ knowledge, only articles in the following languages were included: English, German, French, Italian, and Spanish. Only studies classified as I to III in their level of evidence according to the 2020 Oxford Centre of Evidence-Based Medicine [39] were included. Reviews, letters, editorials, and opinions were excluded. Studies involving in vitro and animal experiments, computational analyses, biomechanical assessments, or cadaveric research were also disregarded. Finally, only studies with a minimum follow-up of 24 months were considered.

### Search strategy

The present systematic review followed the guidelines defined in the 2020 Preferred Reporting Items for Systematic Reviews and Meta-Analyses (PRISMA) statement [40]. The literature investigation followed the PICOTD algorithm:

- P (problem): FAI
- I (intervention): arthroscopy
- C (comparison): torn vs intact ligamentum teres
- (outcomes): PROMs
- T (timings): minimum 24 months of follow-up
- D (design): clinical study.

The Web of Science, PubMed, and Embase were accessed in August 2024 without additional filters or temporal constraints. The medical subject headings (MeSH) used in the research are detailed in the Appendix.

### Selection and data collection

Two authors (F.C. and T.B.) independently conducted the search. All the titles underwent manual screening, and their abstracts were reviewed if deemed relevant. Full texts were singularly scrutinised for further articles matching the inclusion criteria. Articles lacking accessible full texts were excluded. Furthermore, the bibliographies of the full-text articles were cross-referenced for potential inclusion. Any discrepancies between authors were resolved by a third senior author (N.M.), who made the final decision.

### Data items

Data extraction was conducted independently by two authors (F.M. and T.B.). The following items were considered for each study: author, year of publication, journal, study design, and length of follow-up. The following data at the baseline were extracted: number of patients, female sex, and body mass index (BMI). Data concerning the Visual Analogue Scale (VAS) [41], modified Harris Hip Score (mHHS) [42], Hip Outcome Score—activities of daily living (HOS-ADL) [42–44], and Modified Hip Outcome Score—sport-specific subscale (HOS-SSS) [42–44] were assessed both at the baseline and at the last follow-up. Data concerning the following complications were retrieved: revision and progression to total hip arthroplasty (THA). Extraction was performed using Microsoft Office Excel version 16.0 (Microsoft Corporation, Redmond, USA).

### Risk of bias assessment

The risk-of-bias evaluation followed the guidelines described in the* Cochrane Handbook for Systematic Reviews of Interventions* [45]. The risk of bias in the selected articles was independently assessed by two authors (F.C. and T.B.). To analyse the risk of bias in non-randomised controlled trials (non-RCTs), the Risk of Bias in Non-randomised Studies of Interventions (ROBINS-I) tool [46] was used. The tool considers seven domains of potential bias. These domains include bias due to confounding factors and patient selection characteristics before the comparative intervention, bias in the classification of interventions, and bias in the methodological quality when comparing post-intervention outcomes (comprising deviations from intended interventions, missing data, outcomes measurement, and bias in the selection of the reported results). The chart of ROBINS-I was generated using the Robvis software (Risk-of-bias visualization, Riskofbias.info, Bristol, UK) [47].

### Synthesis method

The main author (F.M.) performed the statistical analyses following the recommendations of the Cochrane Handbook for Systematic Reviews of Interventions [45]. Descriptive statistics were calculated using the IBM SPSS software version 25 (International Business Machines Corporation, Armonk, USA). The arithmetic mean and standard deviation were used for continuous data, and the frequency (events/observations) for dichotomic variables.

## Results

### Study selection

**Fig. 1:**
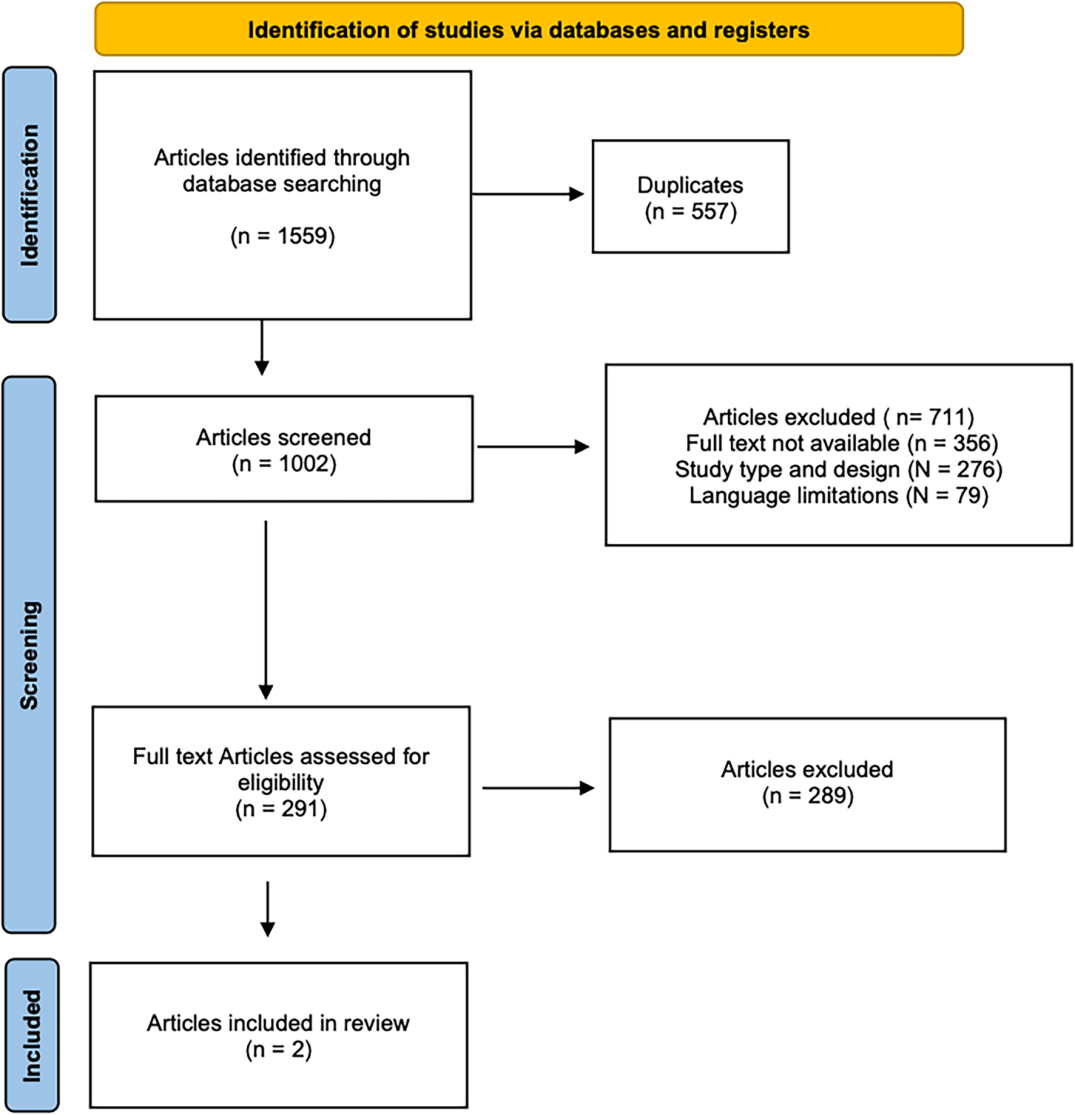
PRISMA flow chart of the literature research

The systematic literature research identified 1559 articles. After removing duplicates, the abstracts of 1002 articles were screened for eligibility. A total of 711 articles were excluded for the following reasons: mismatch with the predefined study design criteria (*N* = 276), full-text unavailability (*N* = 356), and language limitations (*N* = 79). Of the remaining 291 studies, another 289 were excluded after full-text evaluation. Consequently, only two studies were included in this systematic review. The results of the literature search are shown in Fig. 1.

### Risk of bias assessment

**Fig. 2:**
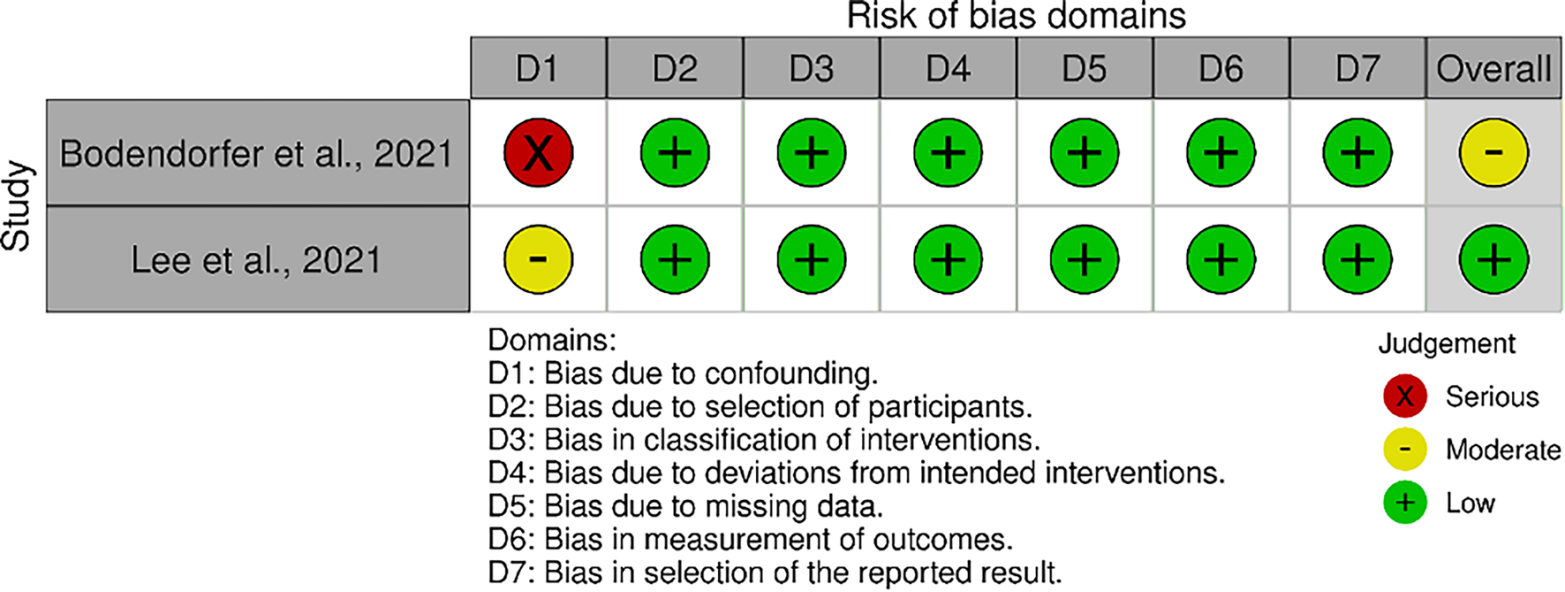
The ROBINS-I of non-RCTs

The ROBINS-I tool was employed to evaluate the risk of bias in the selected non-RCTs (two articles). One article showed a serious risk of bias due to confounding, while the other demonstrated a moderate risk in this domain. Neither article raised concern in the remaining six domains. In conclusion, the ROBINS-I assessment indicated an overall moderate risk of bias for one study and a low risk for the other (Fig. 2).

### Study characteristics and results of individual studies

Data from 611 patients were retrieved, 56.3% of whom (344 of 611) were females. The mean length of follow-up was 45.1 ± 42.9 months. The mean age was 37.4 ± 1.5 years, and the mean BMI was 24.0 ± 0.6 kg/m^2^. In both studies, the torn LT was debrided.

The characteristics of the included studies and patient demographics are shown in Table 1.

**Table 1:** Study details and patient demographics of the included studies

Author and year	Journal	Study design	Ligamentum teres status and treatment	Follow-up (months)	Patients (*n*)	Female sex (*n*)	Mean age (y)	Mean BMI
Bodendorfer et al. 2021 [31]	*Orthop J Sports Med*	Retrospective	Intact	24.5	372	231	37.7	24.3
			Torn (debridement)	24.5	124	77	38.9	24.4
Lee et al. 2021 [48]	*J Hip Preserv Surg*	Retrospective	Intact	131.5	28	9	36.5	22.7
			Torn (debridement)	135.0	87	27	34.0	22.8

### Baseline comparability

The mean length of follow-up, mean age, mean BMI, female/male ratio, VAS, mHHS, HOS-ADL, and HOS-SSS showed baseline comparability between the torn and intact ligament groups (Table 2).

**Table 2:** Baseline comparability

Endpoint	Torn ligament (*N* = 152)	Intact ligament (*N* = 459)	*P*
Female sex	56.6% (86 of 152)	56.2% (258 of 459)	0.98
Follow-up (mean ± SD; months)	44.2 ± 41.6	45.4 ± 43.4	0.5
Age (mean ± SD)	38.5 ± 0.9	37.0 ± 1.5	0.3
BMI (mean ± SD)	24.1 ± 0.7	24.0 ± 0.6	0.99
VAS (mean ± SD)	5.4 ± 2.0	5.3 ± 2.1	0.96
mHHS (mean ± SD)	61.1 ± 16.5	62.5 ± 14.1	0.8
HOS-ADL (mean ± SD)	63.9 ± 14.4	65.3 ± 16.8	0.6
HOS-SSS (mean ± SD)	46.9 ± 21.2	46.3 ± 21.4	0.3

*VAS* Visual Analogue Scale, *mHHS* modified Harris Hip Score, *HOS-ADL* Hip Outcome Score—activities of daily living,* HOS-SSS* Hip Outcome Score—sport-specific subscale

### Synthesis of the PROMs results at the follow-up

No statistically significant difference in VAS, mHHS, HOS-ADL, and HOS-SSS between the groups was found at follow-up (Table 3).

**Table 3:** Results of PROMs

Endpoint	Torn ligament (*N* = 152)	Intact ligament (*N* = 459)	Effect size	*P*
VAS (mean ± SD)	1.7 ± 2.1	2.1 ± 2.1	− 0.4	0.5
mHHS (mean ± SD)	86.6 ± 13.9	85.1 ± 15.1	1.6	0.1
HOS-ADL (mean ± SD)	88.1 ± 11.5	87.0 ± 14.4	1.1	0.7
HOS-SSS (mean ± SD)	76.7 ± 20.6	77.8 ± 23.7	− 1.1	0.5

*VAS* Visual Analogue Scale, *mHHS* modified Harris Hip Score, *HOS-ADL* Hip Outcome Score—activities of daily living,* HOS-SSS* Hip Outcome Score—sport-specific subscale

## Discussion

This systematic review underlines that in patients who undergo hip arthroscopy for FAI, the presence of either an intact or torn ligamentum teres treated with debridement does not influence the postoperative clinical outcomes. At approximately 45 months of follow-up, no statistically significant difference in pain was found according to the modified Harris Hip Score (mHHS) and the activities of daily living and sports subscales of the Hip Outcome Score (HOS) between the two groups.

The LT originates from the transverse acetabular ligament along the inferior margin of the acetabulum [1, 2, 4, 15, 17, 49–52]. Although two different fascicles have been classically described—those which connect posteriorly to the ischial and anteriorly to the pubic side of the acetabular notch on the periosteum—recent cadaveric studies showed that the LT has seven distinct attachments [1, 4, 53]. Specifically, there are six attachment points on the acetabular side that hook the acetabular notch and cotyloid fossa and one on the femur [1, 4, 53]. The fovea capitis is the femoral attachment of the LT; it has an oval shape where the LT fibres converge together [1, 2, 4, 53–55]. With an average length of 30–35 mm and an average cross-sectional diameter of 30.6–59 mm [2, 53–55], the LT is vascularised by the obturator artery and, in some patients, also from a branch of the medial circumflex femoral artery [2, 56, 57]. Moreover, from the age of 8 or 9 up to the end of puberty, the LT is the primary nourishment of the femoral head; after that, it contributes minimally to the proximal femoral blood supply [4, 56, 57]. The obturator nerve (L2–L4) supplies the LT, and, in addition, the ligament presents mechanoreceptors and free nerve endings with proprioceptive and nociceptive properties along its course [2, 58]. Several biomechanical studies on animal models have pointed out the critical role of the LT [2, 4, 7, 8, 10, 12, 59–61]. Static and dynamic stabilisers guarantee hip stability [2, 10, 61–63]. The first group consists of the ball and socket structure of the hip joint, the labrum and the LT, and the dense ligaments and capsule surrounding the joint [2, 10, 61–63]. The LT is strongly activated in hip flexion and external rotation, such as in the squatting position or when the hip is extended and internally rotated [2, 4, 5, 7, 8, 10, 61–63]. These two postures impose the maximum tension on the LT, and cadaveric studies showed that the LT limits hip motion at an average of approximately 73° of abduction, 64° of medial rotation, and 58° of lateral rotation [10, 61–64]. Conversely, the dynamic stabilisers are muscles such as the gluteus medius and minimus, which actively press the femur head into the acetabular socket [2, 61–65]. Meanwhile, the labrum and muscle tension produce negative pressure, sealing the femur head into the acetabulum [66]. Therefore, damage or alteration to the labrum or femoral head results in loss of the negative sealing effect, leading to hip microinstability [11, 66, 67]. The biomechanical role of the LT is paramount in patients with bony instability, such as inferior acetabular insufficiency, borderline or frank hip dysplasia, or some forms of FAI [2, 7, 12, 19, 23, 52, 55]. An average load failure of 204 N characterises the LT [60]. Register et al. [68] evidenced the presence of asymptomatic LT lesions in less than 2.2% of the cohort assessed with a magnetic resonance of 3 T. On the other hand, different data about the prevalence of LT tears, which was found to range from 30 to 90% of HA patients, have been reported in the HA literature [2, 25, 35, 69].

The diagnosis of an LT tear is challenging. The most important risk factors for developing LT tears are modifiable or non-modifiable [2, 16, 35]. The most significant modifiable aspect is high-energy traumatic physical activity, while the non-modifiable factors are an abnormal hip anatomy, female gender, and ligamentous laxity [2, 16, 35]. Domb et al. [16] highlighted a correlation between hip morphology, patient age, and LT lesions. LT tears were more common in patients with a low lateral coverage index and less acetabular anteversion because insufficient acetabular coverage might compromise the joint structural stability and perhaps even the labral seal [16, 35]. Clinically, the most common symptoms are localised groin pain, hip instability, a restricted range of motion, and pain with log hip rolling and passive internal rotation at 90° of flexion. Recently, a “ligamentum teres test” or “O’Donnell’s test” has been proposed [30, 70]. This test consists of reported pain hip pain at extremes of internal and external rotation with the hip in 70° of flexion and 30° from full abduction, and it presents 90% sensitivity and 85% specificity in assessing ligamentum teres tears [14]. Although conventional magnetic resonance (MR) is mostly adequate for investigating a joint, the most reliable radiological investigations to detect LT lesions are magnetic resonance arthrography (MRA) and computed tomography (CT) arthrography [4]. The superiority of these two methods results from the flow of contrast medium between the individual structures, permitting the outline of margins and surfaces depicting the possible chondral and non-chondral lesions in the hip. Chang et al. [28] reported an overall accuracy of 95% in diagnosing LT tears using 1.5-T MRI with arthrography. However, the gold standard for the diagnosis of LT pathology is HA.

Another condition associated with an LT tear is the presence of FAI [19, 23, 25, 52, 55, 71]. The primary symptom of FAI is motion-related or position-related pain in the hip or groin [72–74]. The diagnosis of FAI with or without LT tears is challenging because, commonly, the insidious onset is characterised by chronic pain and a reduced range of motion in the hip without a history of trauma, and there is no single clinical test that allows a clear diagnosis [75–78]. In addition to pain, patients may also describe clicking, catching, locking, and stiffness in the affected hip [79, 80]. Typical imaging modalities for FAI are radiography, MR imaging, and direct MRA [81]. Other methods to determine FAI are less useful: CT allows the visualisation of the bone morphology but does not display lesions of the cartilage or labrum. The role of ultrasound in FAI is not yet established [82]. High-quality anteroposterior pelvis radiographs are the first step in diagnosing FAI. The Dunn 45° view and cross-table lateral view are most useful for the initial assessment [83–86]. On the other hand, MRA demonstrated a higher sensitivity and specificity than conventional MRI regarding hip labrum and cartilage lesions [87]. MRA sensitivity ranges from 85 to 89% and its specificity from 50 to 100% for labrum cartilage; MRA has a sensitivity and specificity for assessing acetabular cartilage ranging from 71 to 92% and from 72 to 85%, respectively [86, 88]. However, the Warwick agreement underlined that specific imaging findings are not always associated with the patient’s symptoms [72]. In this regard, Manner et al. [71] evidenced the crucial impact of FAI on facilitating posterior hip dislocations because engaging cam lesions favour a degree of posterior hip subluxation, causing high strain leading to possible LT tears. Maldonado et al. [19] pointed out a three times greater conversion rate to THA in LT-torn patients [89]. Moreover, degenerative arthritis can promote LT abrasion tears from osteophytes around the edge of the acetabular fossa [2, 15, 19]. Various classifications of LT tears have been proposed (Table 4). Lee et al. [48] enrolled 115 patients to compare clinical and imaging outcomes in patients with cam-type FAI with and without a partial LT tear, dividing the patients into two groups who underwent HA LT tear debridement and femoroplasty with ≥ 10 years of follow-up. Although a significant clinical difference was not observed between patients with an intact LT and those with a torn LT, patients with LT lesions had reduced athletic performance and a higher grade of cartilage damage in both the acetabular region and femoral heads along with worse Tönnis grades [23]. Bodendorfer et al. [32] investigated whether patients with FAI syndrome undergoing HA with labral repair and concomitant LT debridement experienced outcomes similar to patients without LT pathology undergoing only labral repair with a minimum follow-up of 2 years. There were 124 patients with FAI syndrome with a labral tear and a concomitant partial LT tear and 372 patients with a labral tear and no LT pathology [32]. Overall, there was no difference between the groups in pre- and postoperative outcomes and in achieving the minimal clinically important difference (MCID) and the patient-acceptable symptomatic state (PASS) values [32]. Furthermore, Bodendorfer et al. did not observe any complications [32].

**Table 4:** Overview of LT tear classification

Author classification group	LT pathological alteration and grade
Domb classification [68]	I: normal II: partial tears (< 50%) III: partial tears (> 50%) IV: complete tears
Gray and Villar [13]	I: complete rupture (major trauma) II: partial rupture (minor trauma) III: degenerative—partial or complete (attritional)
Salas and O’Donnell [89]	I: LT synovitis II: LT synovitis with impingement III: partial LT tear—low grade IV: partial LT tear—high grade (50%) V: partial LT tears with hip osteoarthritis VIa: complete LT tear—acquired VIb: complete LT tear—avulsion fracture VIc: complete LT tear—congenital absence
O’Donnell and Arora [30]	0: normal I: synovitis II: partial tear III: complete tear

This study has strengths and limitations. Firstly, the LT is a fundamental structure, but little of the literature considers how its status impacts the clinical outcomes in patients undergoing HA for FAI. The increasing interest in HA for FAI could provide a valuable source for surgeons encountering this pathology. However, drawbacks exist. Only two studies were included in the analysis, so the patient cohort was small. Moreover, both studies were retrospective, and data other than patient questionnaires, clinical charts, and imaging were not obtained. The lack of specific information in terms of cam impingement severity, duration of preoperative symptoms or previous activity level, amount of labral debridement, and adequacy of cam deformity correction may result in differences in the cartilage status observed during HA and in the progression to more severe osteoarthritis during the follow-up, with a higher rate of conversion to THA. Moreover, patient-reported outcome measures (PROMs) were recorded in both studies, but PASS and MCID values were calculated in only one study. In addition, the studies differed in their patient selection criteria, which makes standardisation impossible. The two studies had different follow-up durations, making a comparison of treatment durability difficult. LT tears were evaluated using various classification systems. HA is an operator-dependent technique that can be used as a diagnostic and treatment tool. Given the large variability regarding surgical indication, type of surgical treatment, and intraoperative findings, it is not always possible to distinguish between the role of an LT tear as a pain generator and the effect of the surgical treatment on the outcomes. In the future, further standardised studies with more patients will be required, as will accurate standardisation and clarification of inclusion–exclusion criteria, classification of the LT lesion, and, consequently, postoperative outcomes.

## Conclusions

An intact or torn ligamentum teres managed with debridement does not influence the postoperative PROMs in patients undergoing arthroscopic management for FAI.

## Supplementary information

Supplementary Material 1.

## Data Availability

The datasets generated during and/or analysed during the current study are available throughout the manuscript.
