# Supplementary material for: The ligamentum teres and its role in hip arthroscopy for femoroacetabular impingement: a systematic review
Source: J Orthop Traumatol. 2024 Dec 20;25:68. doi: 10.1186/s10195-024-00810-1 (PMC11662112; doi:10.1186/s10195-024-00810-1)
Supplement: Supplementary file 1 — Supplementary Material 1. [file 10195_2024_810_MOESM1_ESM.docx]

**Research Question:**

**Femoracetabular Impingement with or without Ligamentum Teres Tear**

**Concept 1 – Problem: FAI**

**Keywords:**

Acetabulum

cam

Cam impingement

cam lesion

cartilage

chondral lesion

Conflict

FAI

FAI syndrome

Femoracetabular impingement

Femoro-acetabular impingement

impingement

Pincer impingement

**Mesh:**

"Acetabulum"[Mesh]

"Acetabulum / injuries"[Mesh]

"Acetabulum / pathology"[Mesh]

"Cartilage"[Mesh]

"Cartilage / physiopathology"[Mesh]

"Femoracetabular Impingement"[Mesh]

"Femoracetabular Impingement / epidemiology"[Mesh]

"Femoracetabular Impingement / pathology"[Mesh]

"Femoracetabular Impingement / physiopathology"[Mesh]

"Fibrocartilage / physiopathology"[Mesh]

"Hip"[Mesh]

"Hip Joint / pathology"[Mesh]

"Pain"[Mesh]

**Concept 2 - Intervention: Arthroscopy**

**Keywords:**

acetabular labral refixation

Acetabular Rim Resection

arthroscopic labral reconstruction

arthroscopic surgery

Arthroscopy

Debridement

hip arthroscopic surgery

Hip arthroscopy

labral reconstruction

labral repair

labrum repair

Reconstruction

Repair

**Mesh:**

"Acetabulum / surgery"[Mesh]

"Arthroscopy"[Mesh]

"Arthroscopy / methods"[Mesh]

"Arthroscopy / standards"[Mesh]

"Debridement"[Mesh]

"Femoracetabular Impingement / surgery"[Mesh]

"Fibrocartilage / surgery"[Mesh]

"Hip Joint / surgery"[Mesh]

**Concept 3 – Comparison: Torn vs Intact Ligamentum Teres**

**Keywords:**

Round Ligament

Ligamentum Teres

Ligamentum Teres tear

Round Ligament tear

**Mesh:**

"Round Ligament of Femur"[Mesh]

"Round Ligament of Femur / injuries"[Mesh]

"Round Ligament of Femur /pathology"[Mesh]

**Concept 4 - Outcome: PROMS**

**Keywords:**

clinical outcome

Harris Hip Score

hip outcome score

patient outcomes

Patient Reported Outcome Measures

Treatment outcome

VAS

visual analog scale

**Mesh:**

"Patient Outcome Assessment"[Mesh]

"Patient Reported Outcome Measures"[Mesh]

"Patient Satisfaction"[Mesh]

"Quality of Life"[Mesh]

**Searching Strategy**

"Acetabulum"[Mesh] OR "Acetabulum / injuries"[Mesh] OR "Acetabulum / pathology"[Mesh] OR "Cartilage"[Mesh] OR "Cartilage / physiopathology"[Mesh] OR "Femoracetabular Impingement"[Mesh] OR "Femoracetabular Impingement / epidemiology"[Mesh] OR "Femoracetabular Impingement / pathology"[Mesh] OR "Femoracetabular Impingement / physiopathology"[Mesh] OR "Fibrocartilage / physiopathology"[Mesh] OR "Hip"[Mesh] OR "Hip Joint / pathology"[Mesh] OR "Pain"[Mesh] OR Acetabulum OR cam OR Cam impingement OR cam lesion OR cartilage OR chondral lesion OR Conflict OR FAI OR FAI syndrome OR Femoracetabular impingement OR Femoro-acetabular impingement OR impingement OR Pincer impingement

**AND**

"Acetabulum / surgery"[Mesh] OR "Arthroscopy"[Mesh] OR "Arthroscopy / methods"[Mesh] OR "Arthroscopy / standards"[Mesh] OR "Debridement"[Mesh] OR "Femoracetabular Impingement / surgery"[Mesh] OR "Fibrocartilage / surgery"[Mesh] OR "Hip Joint / surgery"[Mesh] OR acetabular labral refixation OR Acetabular Rim Resection OR arthroscopic labral reconstruction OR arthroscopic surgery OR Arthroscopy OR Debridement OR hip arthroscopic surgery OR Hip arthroscopy OR labral reconstruction OR labral repair OR labrum repair OR Reconstruction OR Repair

**AND**

"Round Ligament of Femur"[Mesh] OR "Round Ligament of Femur / injuries"[Mesh] OR "Round Ligament of Femur /pathology"[Mesh] OR Round Ligament OR Ligamentum Teres OR Ligamentum Teres tear OR Round Ligament tear

**AND**

"Patient Outcome Assessment"[Mesh] OR "Patient Reported Outcome Measures"[Mesh] OR "Patient Satisfaction"[Mesh] OR "Quality of Life"[Mesh] OR clinical outcome OR Harris Hip Score OR hip outcome score OR patient outcomes OR Patient Reported Outcome Measures OR Treatment outcome OR VAS OR visual analog scale

**SUMMARY**

("Acetabulum"[Mesh] OR "Acetabulum / injuries"[Mesh] OR "Acetabulum / pathology"[Mesh] OR "Cartilage"[Mesh] OR "Cartilage / physiopathology"[Mesh] OR "Femoracetabular Impingement"[Mesh] OR "Femoracetabular Impingement / epidemiology"[Mesh] OR "Femoracetabular Impingement / pathology"[Mesh] OR "Femoracetabular Impingement / physiopathology"[Mesh] OR "Fibrocartilage / physiopathology"[Mesh] OR "Hip"[Mesh] OR "Hip Joint / pathology"[Mesh] OR "Pain"[Mesh] OR Acetabulum OR cam OR Cam impingement OR cam lesion OR cartilage OR chondral lesion OR Conflict OR FAI OR FAI syndrome OR Femoracetabular impingement OR Femoro-acetabular impingement OR impingement OR Pincer impingement) AND ("Acetabulum / surgery"[Mesh] OR "Arthroscopy"[Mesh] OR "Arthroscopy / methods"[Mesh] OR "Arthroscopy / standards"[Mesh] OR "Debridement"[Mesh] OR "Femoracetabular Impingement / surgery"[Mesh] OR "Fibrocartilage / surgery"[Mesh] OR "Hip Joint / surgery"[Mesh] OR acetabular labral refixation OR Acetabular Rim Resection OR arthroscopic labral reconstruction OR arthroscopic surgery OR Arthroscopy OR Debridement OR hip arthroscopic surgery OR Hip arthroscopy OR labral reconstruction OR labral repair OR labrum repair OR Reconstruction OR Repair) AND ("Round Ligament of Femur"[Mesh] OR "Round Ligament of Femur / injuries"[Mesh] OR "Round Ligament of Femur /pathology"[Mesh] OR Round Ligament OR Ligamentum Teres OR Ligamentum Teres tear OR Round Ligament tear) AND ("Patient Outcome Assessment"[Mesh] OR "Patient Reported Outcome Measures"[Mesh] OR "Patient Satisfaction"[Mesh] OR "Quality of Life"[Mesh] OR clinical outcome OR Harris Hip Score OR hip outcome score OR patient outcomes OR Patient Reported Outcome Measures OR Treatment outcome OR VAS OR visual analog scale)
